# Associations of extended work, higher workloads and emotional work demands with sleep disturbance among night-shift workers

**DOI:** 10.1186/s12889-022-14599-3

**Published:** 2022-11-21

**Authors:** Bo Min Jeon, Su Hyun Kim

**Affiliations:** 1 College of Nursing, Kyungpook National University, Daegu, Korea; 2 College of Nursing, The Research Institute of Nursing Science, Kyungpook National University, 680 Gukchaebosang-Ro, Jung-Gu, Daegu, 41944 Korea

**Keywords:** Employees, Shift work schedule, Social environment, Sleep–wake disorders

## Abstract

**Background:**

In-depth investigation of the factors that exacerbate sleep disturbance among night-shift workers is essential to develop a successful implementation strategy to improve sleep. Although some characteristics of work associated with sleep disturbances have been investigated, there are inconsistencies in the findings. This study aimed to assess the influence of working time and work characteristics on sleep disturbance among night-shift workers.

**Methods:**

This study was a secondary data analysis of a nationally representative sample of data from the fifth Korean Working Condition Survey in 2017. A total of 1,790 wage workers aged between 19 and 64 years with night shift schedules were selected and analyzed. A multiple logistic regression analysis was conducted to analyze the influences of working time characteristics, including shift type, frequency of night shift, and extended work; as well as work characteristics, including physical work demands, workload, emotional work demands, social support, and communication, on sleep disturbance among night-shift workers.

**Results:**

Of those analyzed, 8.4% of night-shift workers experienced sleep disturbances. Night-shift workers with extended work, higher workloads, and emotional work demands were more likely to experience sleep disturbances (OR = 1.53, 95% CI = 1.05 to 2.23; OR = 1.01, 95% CI = 1.01 to 1.03; OR = 1.03, 95% CI = 1.02 to 1.05; respectively).

**Conclusion:**

Extended work, higher workload, and emotional work demands were significant factors for sleep disturbance among night-shift workers. These findings highlight the importance of adjusting work demands and mitigating extended work to reduce sleep disturbance in night-shift workers.

## Background

The number of night-shift workers is increasing with the expansion of 24-h service [1]. In Europe, approximately 20% of all employees have a night-shift work schedule, typically working on 11 p.m. to 7 a.m. or 12 midnight to 8 a.m. [2, 3]. In Korea, 10.4% of employees are engaged in a night-shift work schedule for all businesses [4]. A night-shift work schedule needs more attention because of the increased risk of experiencing sleep disturbance [5–8]. In the literature, night-shift workers had a 2.7 to 3.9 times higher risk of insomnia than day-shift workers [9]. They often reported difficulty falling asleep and drowsiness at work [10, 11] and had a higher prevalence of sleep–wake disorders than day-shift workers [6]. Night-shift workers have an increased risk of cardiovascular diseases, diabetes, and obesity [12]. In-depth investigation of the factors that exacerbate sleep disturbance among night-shift workers is essential to develop successful implementation strategies to improve the sleep and health of night-shift workers.

According to the comprehensive model on nonstandard working schedules and health [13], working time and work characteristics influence shift workers’ health through physiological processes. In the model, work is defined as the period in which a person spends physical and mental effort to perform tasks required in the workplace. Important keys for work are featured with the working time characteristics of work, such as time-of-day, working time duration, and type of shifts; and work characteristics, such as work demands, and psychosocial context of work [13]. These characteristics, alone or in combination, may lead to adverse health effects through the physiological processes of circadian disruption, sleep deprivation, and increased or sustained activation [13]. In the literature, the work characteristics influencing sleep disorders of night shift workers included a rotating shift schedule [14, 15], longer working hours [9], higher workload, work demands, and lower compensation [16]. Other characteristics of the psychosocial context of work, such as social support, control, and level of organizational justice, have also been associated with the severity of sleep disorders [16].

To prevent sleep disturbance in night-shift workers, more attention needs to be paid to identify the impact of working time and work characteristics, as indicated in the comprehensive model on nonstandard working schedules and health [13, 16]. Although a few previous studies have investigated some characteristics of work associated with sleep problems among employed workers, there have been inconsistencies in the findings [16]. Moreover, the findings regarding the characteristics of work associated with sleep disturbance were not specified for night-shift workers, which complicates the understanding of the influence of work characteristics on sleep disturbance for night-shift workers, who are the most vulnerable work population. Though previous studies have investigated how shift work and day work affect sleep disturbance [17], it is imperative to identify the work characteristics that predict sleep disturbance among night-shift workers in order to develop strategies that can help improve their sleep quality despite their nonstandard working schedules [13].

Therefore, this study was conducted to gain a comprehensive understanding of the influence of working time and work characteristics on sleep disturbance among night-shift workers. Particularly, by analyzing a nationally representative sample of Korean workers, the Fifth Korean Working Conditions Survey (KWCS) (2017), this study aimed to identify the prevalence and influence of the characteristics of working time and work on night-shift workers in South Korea. Based on a comprehensive theoretical model of nonstandard working schedules and health [13], it was hypothesized that sleep disturbance among night-shift workers is influenced by the characteristics of working time (shift type, frequency of night work, and extended work) and work (physical work demands, workload, emotional work demands, social support, and communication) after adjusting for demographic characteristics (sex, age, education, and income).

## Methods

### Study population

This study was a secondary data analysis that utilized data obtained from the Fifth KWCS of 2017, conducted by the Occupational Safety and Health Research Institute (Approval No. 380002) [18]. The original data were those of 50,000 employees aged between 19 and 65 years, extracted from a 2010 Population Census Data, using a multi-stage probability proportion stratified cluster sampling method [18]. After selecting census districts using the probability proportional sampling method, 10 households were randomly selected by systematic sampling in each selected census district. One eligible person in each household engaged in the labor market was randomly selected for interviews. The original data were collected through a 1:1 face-to-face interview at the respondents’ residence from July to November 2017. Data that could identify the unique personal information of the survey participants were not collected.

The inclusion criteria of the participants in this study were: i) “wage worker;” ii) those with work type of “shift work;” iii) those who had at least one-night shift per month; and iv) those aged from 19 to 64 years. The age limit of under 65 was applied to exclude the influence of the aging process on sleep disturbance, such as changes in melatonin secretion and circadian cycle imbalance [19]. Workers aged less than 19 years were excluded to avoid confounding effects of developmental processes on sleep disturbance [20]. A total of 1,790 participants who met the inclusion criteria were included in this study. This study was approved by the Bioethics Review Committee of the affiliated institution. This study was carried out in accordance with relevant guidelines and regulations (e.g. Declaration of Helsinki).

### Variables

Sleep disturbance were assessed using the Minimal Insomnia Symptom Scale (MISS), which comprised three items: “Within the last 12 months, how often did you experience problems with: i) having difficulties in falling asleep; ii) repeatedly waking up during sleep; iii) waking up feeling tired or exhausted?” [21]. The responses were answered on a numerical scale, which ranged from “every day” (1 points), “several times a week” (2 points), “several times a month” (3 points), “rarely” (4 points), and “not at all” (5 points). The responses “don’t know” and “decline” were coded as non-responses. For this study, the scores were reverse-coded and summed to obtain the total score, with higher scores indicating severe insomnia symptoms. Cronbach’s alpha coefficient was 0.73 in a previous study (Broman et al., 2008) and 0.75 in this study. A cutoff score ≥ 6 was suggested to be optimal for discriminating sleeping difficulty, with a sensitivity of 0.82 and specificity of 0.86, with International Classification of Disease (ICD-10) research criteria for insomnia as a criterion standard in a population of 20–64 years [21].

The characteristics of working time, “shift type,” “extended work,” and “frequency of night work” were assessed using a questionnaire. First, responses to the question regarding shift type, “Which of the following best represents the type of your shift work?,” were as follows: split shifts, shift/rotation, and permanent shifts. A split shift refers to a work schedule that divides a working day into two or more working periods separated by periods of break time, which require a shift interval of at least four hours per workday. For example, an employee works their first shift from 04:00 to 08:00, takes a break until 13:00, and then works their second shift from 13:00 to 17:00. Rotating shifts refer to work schedules that shift alternately between day, evening, and night work according to a predetermined rotating schedule. Second, extended work was assessed by asking, “How many days per month do you work more than 10 h a day?” If participants recorded the number of days, they were coded as an extended work group; if not, they were coded as a nonextended work group. Third, the frequency of night work was measured by the number of days each participant responded to the question, “How many night shifts (working at least two hours between 10 pm and 5 am) do you work per month?”.

Work characteristics, physical work demands, workload, emotional work demands, social support, and communication were assessed using questionnaires. Both physical work demands and workload were assessed with five items and four items respectively, on a 7-point Likert scale ranging from “throughout working hours” to “not exposed at all”. An example item for physical work demands is, “Does your work involve tiring or painful postures?” and for workload, “How often does your work require working at fast speed?.” The total scores were calculated by summing up each response to the questions and converting them to a score of 0–100; a higher total score indicated a higher physical work demand and workload. Cronbach’s alpha coefficient of the scales ranged from 0.75 to 0.80 [20] and were 0.64 in this study. Emotional work demands were measured with four questions: “Does your work involve complicated tasks?”, “Does your work deal with angry customers or patients?”, “Does your work place you into being emotionally unstable?” and “I need to hide my emotion in my working place.” The responses on dichotomous, or 5- or 7-point Likert scales for each question were converted to a score of 0–100 after summing the scores; with higher scores indicating higher levels of emotional work demands. Cronbach’s alpha coefficient for the scales was 0.71 [22] and was 0.50 in this study.

Social support was measured using three items: “My colleagues help and support me.”, “My boss helps and supports me.”, and “We are fairly treated in the workplace.” The responses on a 5-point Likert scale ranging from “always” to “not at all” were converted to a score of 0–100 after summing the scores. Cronbach’s alpha coefficient of the scales ranged from 0.76 to 0.85 [20] and was 0.66 in this study. Communication was measured with six items on a 5-point Likert scale (from “strongly agree” to “strongly disagree”). Example items are “Employees are recognized and complimented for good work in the workplace.” and “My colleagues and I are in good cooperation.” Responses were summed and converted to a score of 0–100, with higher scores indicating higher levels of social support. Cronbach’s alpha coefficient of the scales ranged from 0.68 to 0.87 [22] and was 0.86 in this study. General characteristics included gender, age, level of education, and monthly income.

### Statistical analysis

As the work environment survey was based on a complex sample design, weighting was applied when analyzing the data to accurately estimate the population distribution [18]. Differences in sleep disturbance based on general characteristics, as well as work time and work characteristics, were analyzed using an independent t-test or χ^2^ test. The proportion of missing values was less than 1%, except the variables of communication (5.6%), income (4.1%), and social support (2.9%). No systematic pattern was observed for the missing values of the variables, and multiple imputations were conducted for missing values using the SPSS software. Missing values were replaced with a random sample of plausible value imputations via single-value regression analysis [23]. Five imputed datasets were created and the results obtained from each completed data analysis were pooled into a single multiple imputation result.

Multiple hierarchical logistic regression analysis was conducted to explore the influence of working time characteristics and work characteristics on sleep disturbance after accounting for demographic characteristics. The odds ratios (OR) and 95% confidence intervals were calculated for all models. Model 1 included demographic characteristics, such as gender, age, education, and income level. In Model 2, working time characteristics, such as shift type, frequency of night shifts per month, and extended work were added to identify the impacts of working time characteristics over and above demographic characteristics. In Model 3, work characteristics, such as physical work demands, physical workload, emotional work demands, social support, and communication, were added to identify the additional impacts of working characteristics over and above working time and demographic characteristics. Demographic variables, such as gender, education, and income level, were analyzed as categorical variables. Sex was classified into two groups (male and female). Education level was classified into three groups (middle school graduate, high school graduate, and college graduate or higher education). Income level was classified into four groups (USD 1300 or less, 1300–2500, 2500–4000, and 4000 or more), and age was analyzed with a continuous variable. We used a *p*-value of < 0.05 to determine statistical significance. SPSS/WIN 25.0 software was used for data analysis.

## Results

### Participants’ characteristics

Participants’ characteristics are presented in Table 1. Approximately 75% of the participants were male, and the mean age was 42.5 ± 12.6 years. Overall, 62.2% of the participants worked in rotating shifts, and 46.5% had extended work. A total of 26.9% were exposed to rotating shifts and extended work, 8.4% to split shifts and extended work, and 10.7% to fixed shifts and extended work. The monthly mean frequency of night-shift work and extended work was 10.1 ± 5.0 days and 6.0 ± 8.2 days, respectively. Regarding work characteristics, the participants had a mean of 37.2 ± 16.0 for physical work demands and 45.5 ± 16.4 for workload. They had a mean of 40.9 ± 16.2 for emotional work demands, 68.0 ± 17.0 for social support, and 65.2 ± 13.3 for communication.

**Table 1:** Demographic and work environment characteristics of the participants (*N* = 1790)

Characteristics	Total (*N* = 1790)	No sleep disturbance (*N* = 1640)	Sleep disturbance (*N* = 150)	χ^2^ or t (*p*)
	n (%) or mean ± SD			
**Demographic characteristics**				
Gender				
Male	1338 (74.8)	1232 (75.1)	106 (71.1)	1.1 (.284)
Female	452 (25.2)	408 (24.9)	44 (28.9)	
Age (years)	42.5 ± 12.6	42.3 ± 12.6	43.6 ± 12.2	1.2 (.244)
Education				
Middle school	94 (5.3)	90 (5.5)	4 (2.7)	3.5 (.178)
High school	809 (45.2)	733 (44.7)	76 (50.6)	
College or more	887 (49.5)	817 (49.8)	70 (46.7)	
Monthly income (USD)				
≤ 1300	180 (10.1)	161 (9.8)	19 (12.6)	5.7 (.125)
1300 ~ 2500	849 (47.4)	768 (46.8)	81 (54.0)	
2500 ~ 4000	560 (31.3)	526 (32.1)	34 (22.7)	
≥ 4000	201 (11.2)	185 (11.3)	16 (10.7)	
**Working time characteristics**				
Shift type				
Split shifts	292 (16.3)	266 (16.2)	26 (17.3)	2.8 (.243)
Fixed shifts	384 (21.5)	345 (21.0)	39 (26.0)	
Rotating shifts	1114 (62.2)	1029 (62.8)	85 (56.7)	
Frequency of night-shift (per month)	10.1 ± 5.0	10.1 ± 5.0	10.2 ± 5.3	0.1 (.913)
Extended work				
Yes	833 (46.5)	748 (45.6)	85 (56.7)	6.8 (.009)
No	957 (53.5)	892 (54.4)	65 (43.3)	
Frequency of extended work (per month)	6.0 ± 8.2	6.0 ± 8.2	6.8 ± 8.4	1.2 (.212)
**Work characteristics**				
Physical work demands	37.2 ± 16.0	36.8 ± 15.6	41.6 ± 19.5	2.9 (.003)
Workload	45.5 ± 16.4	39.1 ± 16.2	44.2 ± 17.9	3.4 ( .001)
Emotional work demands	40.9 ± 16.2	40.0 ± 15.4	50.2 ± 20.7	5.9 (< .001)
Social support	68.0 ± 17.0	68.1 ± 17.0	66.4 ± 16.9	-1.1 (.268)
Communication	65.2 ± 13.3	65.4 ± 12.9	62.3 ± 16.3	-2.3 (.023)

### Sleep disturbance according to demographic, working time and work characteristics

Among the participants, 8.4% (n = 150) experienced sleep disturbances. Bivariate analyses (Table 1) showed no differences in the presence of sleep disturbance according to demographic characteristics, such as gender, age, education level, and income level. In terms of working time characteristics, there were no differences in shift type and frequency of night shifts per month and extended work. However, workers with extended work were more likely to experience sleep disturbances (χ^2^ = 6.8, *p* = 0.009). Regarding work characteristics, workers exposed to physical work demands (t = 2.9, *p* = 0.003), workload (t = 3.4, *p* = 0.001) and emotional work demands (t = 5.9, *p* < 0.001) experienced more sleep disturbances. In contrast, workers who communicated effectively with colleagues and managers had fewer sleep disturbances (t = -2.3, *p* = 0.023).

### Influences of working time and work characteristics on sleep disturbance

In the hierarchical multiple logistic regression analysis (Table 2) in Model 1, the odds of developing sleep disturbance increased with high school education or college education in comparison to middle school education (OR = 2.95, 95% CI = 1.13 to 8.26; OR = 3.00, 95%  CI= 1.12 to 8.88, respectively). In Model 2, the odds of developing sleep disturbance significantly increased when the participants had extended work (OR = 1.60, 95% CI = 1.11 to 2.31), adjusting for gender, age, education, and income level. In Model 3, higher workloads and emotional work demands were significantly associated with increased odds of sleep disturbance (OR = 1.01, 95% CI = 1.01 to 1.03; OR = 1.03, 95% CI = 1.02 to 1.05, respectively) in addition to extended work (OR = 1.53, 95% CI = 1.05 to 2.23). Shift type, frequency of night-shift per month, physical work demands, social support, and communication were not significantly associated with sleep disturbance after adjusting for demographic characteristics.

**Table 2:** Influence of demographic and work environment on sleep disturbance (*N* = 1790)

Variables	Model 1		Model 2		Model 3	
	OR	95% CI	OR	95% CI	OR	95% CI
**Demographic characteristics**						
Age	1.01	1.00–1.03	1.01	1.00–1.03	1.01	0.99–1.02
Gender						
Male	ref		ref		ref	
Female	1.18	0.79–1.77	1.34	0.90–2.10	1.16	0.74–1.81
Monthly income (USD)						
≤ 1300	ref		ref		ref	
1300 ~ 2500	0.83	0.46–1.49	0.83	0.45–1.52	0.79	0.42–1.47
2500 ~ 4000	0.49	0.24–1.01	0.52	0.25–1.08	0.52	0.24–1.12
≥ 4000	0.60	0.28–1.30	0.66	0.30–1.47	0.76	0.33–1.75
Education						
Middle school	ref		ref		ref	
High school	2.95^*^	1.13–8.26	3.17^*^	1.13–8.90	2.40	0.84–6.83
College or more	3.00^*^	1.12–8.88	3.32^*^	1.12–9.81	2.47	0.81–7.52
**Working time characteristics**						
Shift type						
Split shifts			ref		ref	
Fixed shifts			1.23	0.70–2.16	1.54	0.85–2.80
Rotating shifts			0.88	0.54–1.43	0.92	0.56–1.52
Frequency of night-shift (per month)			0.99	0.96–1.03	0.98	0.94–1.02
Extended work						
No			ref		ref	
Yes			1.60^*^	1.11–2.31	1.53^*^	1.05–2.23
**Work characteristics**						
Physical work demands					1.01	0.99–1.02
Workload					1.01^*^	1.01–1.03
Emotional work demands					1.03^*^	1.02–1.05
Social support					1.01	0.99–1.01
Communication					0.99	0.97–1.00

*OR* odds ratio, *95% CI* 95% confidence interval

^*^p < .05, ^**†**^Adjusted for gender, age, education, and income level as covariates

## Discussion

In this study analyzing a representative sample of Korean workers in the 2017 KWCS, 8.4% of night-shift wage workers aged between 19 to 64 had sleep disturbance. Extended work and higher workload and emotional work demands were significant factors influencing sleep disturbance among night-shift workers. The prevalence of sleep disturbance in this study was far lower than the one in the previous study utilizing the data of the 2017 KWCS, which reported 52.2% of male and 52.9% of female wage workers having “unstable” sleep conditions [24]. However, the prevalence of sleep disturbance in this study was comparable to the combined prevalence of moderate insomnia (6.6%) and severe insomnia (1.5%) in night-shift workers in manufacturing plants measured using the Insomnia Severity Index (ISI) [25]. It is assumed that the proportion of sleep disturbance in this study presents a more accurate figure for night-shift wage adult workers because it was estimated from national representative data with criteria using the validated cutoff score of the MISS [21].

Among the working time characteristics, “extended work” had the most significant influence on sleep disturbance, where the odds of sleep disturbance were approximately 1.5 times higher for night-shift workers who worked more than 10 h a day than for those who did not. This was in line with previous studies that identified a significant influence of extended work on the prevalence of severe drowsiness in night-shift workers [11] and insomnia in all wage workers [9]. This finding supports the idea that extended work leads to increased activation of the body’s physiological systems, which may lead to sleep disturbance when the increased activation is sustained until bedtime [13]. Extended work may also affect the hours of recovery from tiredness and personal leisure time that counteract the increased activation from work, which in turn has a negative influence on sleep [26, 27]. Thus, it is necessary to ensure optimal working hours for night shift workers, and in cases where extended work is inevitable, it is important to allocate sufficient time for recovery activities [13].

However, the mean days of night-shift per month were 10.1 ± 5.0 days in this study, but no significant influence on sleep disturbance was identified. This finding is in agreement with a previous study that reported that the frequency of night shifts per month was not significantly associated with sleep disturbance among night-shift nurses [28]. One of the possible reasons for the lack of association with frequency of night shift might be that once circadian rhythm is disrupted from night-shift work, it has a greater impact on sleep disturbance than the frequency of night-shift does [28]. Further investigation is warranted to identify the influence of diverse shift types, such as direction and speed of rotating shifts, in combination with frequency of night shifts with objective sleep assessment on sleep disturbance among night-shift workers.

Among the work characteristics, “workload” and “emotional work demands” were the significant factors influencing sleep disturbance, which increased the odds of sleep disturbance by 1–3%, as one unit of workload as well as emotional work demands increased on a 0–100 scale. These findings were consistent with a previous study that identified a significant association between workload, emotional demands, and sleep disturbance in hospital nurses in France [29] and Korea [30], and wage workers in Sweden [31] and France [32]. As high work demands are a typical workplace stressor [33], resulting in increased activity of the sympathetic nervous system and subsequent sleep disturbance [13, 34], it is necessary to adjust unfavorable work demands to improve sleep disturbance in night-shift workers. For example, it might be necessary to develop a proper management plan to reduce the frequency and intensity of workload in night-shift workers [35] and implement organizational work protocols to reduce difficulty at work [13]. In addition, for night-shift workers who work in emotionally unstable situations, such as dealing with confrontational customers or hiding emotions at work, interventions to support positive coping strategies at work or after work hours are required [13].

Social support and communication did not have a significant influence on sleep disturbance. This was in agreement with a previous study on hospital nurses in Korea, in which social support from colleagues or supervisors, and trust and justice at the workplace did not influence sleeping troubles [30]. Although further investigation is needed to identify discrepancies in the influence of social support and communication on sleep disturbance, it seems that social support and communication might have an indirect rather than a direct influence on sleep disturbance among all wage workers [30]. In the literature, it is suggested that support from superiors and colleagues may help manage conflict at work and subsequently mitigate adverse impacts on workers’ health [36], so further investigation about the mediating or moderating effects on sleep and health among night-shift workers is needed.

This study had a few limitations. As the data were collected based on self-reports, it was difficult to exclude self-report bias from this study. Additionally, as the primary purpose of the original data was to understand the influence of the work environment on workers’ health in Korea, detailed information about specific occupations could not be investigated in this study. As work characteristics may have different impacts on sleep according to occupation type, gender, and age, further studies are required for each industry involving night-shift work as well as the modification effect of occupation type, gender, and age. Cronbach’s alpha coefficient to assess emotional work demands was insufficient, but it seemed to be attributed to the small number of items in the instrument [37]. Nevertheless, this study has a strength of having used nationally representative data from adult workers in Korea to investigate the comprehensive working time and work characteristics associated with sleep disturbance in night-shift workers [13].

## Conclusion

Sleep disturbances in night-shift workers are affected by extended work, workload, and emotional work demands. As sleep disturbance of night-shift workers may lead to adverse health outcomes and any occupational hazard, countermeasures to prevent sleep disturbance are required, such as adjusting the working time, workload, and emotional work demands for night-shift workers.

## Data Availability

The datasets used and/or analyzed during the current study are available from the corresponding author upon reasonable request.

## Abbreviations

KWCS: Korean Working Conditions Survey
CI: Confidence interval
